# Postoperative Atrial Fibrillation Following Anatomic Lung Resection: A Case Report and Narrative Review of Contemporary Pharmacologic Management

**DOI:** 10.7759/cureus.89426

**Published:** 2025-08-05

**Authors:** Jordan Sauve, Colin R Tang-Whitmore, Zoe Randall, Pravin Meshram, Sean H Nguyen, Anmol Nigam, Milind Bhagat, Jonathan Berger, Ilitch Diaz-Gutierrez, Diana Oramas Mogrovejo, James V Harmon

**Affiliations:** 1 Department of Surgery, University of Minnesota, Minneapolis, USA; 2 Department of Medicine, University of Minnesota, Minneapolis, USA; 3 Department of Laboratory Medicine and Pathology, University of Minnesota, Minneapolis, USA

**Keywords:** anatomic lung resection, atrial fibrillation, postoperative complication, pulmonary lobectomy, rate control, rhythm control, segmentectomy

## Abstract

Postoperative atrial fibrillation (POAF) is a common complication following anatomic lung resection, contributing to increased morbidity and mortality, prolonged hospital stays, and higher healthcare costs. Despite its frequency, there remains limited consensus on optimal pharmacologic management in this population, particularly in the context of balancing efficacy with the unique risks associated with thoracic surgery. This report aims to draw attention to the clinical significance of POAF in thoracic surgery, particularly following pulmonary resections, by presenting a representative case and contextualizing it through a focused review of current literature and consensus guidelines. A 77-year-old man developed POAF with rapid ventricular response (RVR) following a uniportal video-assisted thoracoscopic surgery (VATS) right lower lobe (RLL) basilar segmentectomy. The patient’s clinical course, therapeutic interventions, and outcomes are discussed in the context of existing guidelines and treatment paradigms. To contextualize the case, we conducted a literature review focused on pharmacologic management strategies for POAF following anatomic lung resection, specifically pulmonary lobectomy. We reviewed PubMed and Scopus databases, applying predefined inclusion and exclusion criteria to identify relevant peer-reviewed articles. Included articles were peer-reviewed studies evaluating pharmacologic treatments for adult patients with new-onset POAF following pulmonary lobectomy. Studies were excluded if they involved only non-lobectomy surgeries, patients with pre-existing atrial fibrillation (AF) or atrial flutter, did not discuss treatment interventions, or included fewer than 10 patients. Our review highlights the evidence supporting commonly used agents such as amiodarone, beta-blockers, calcium channel blockers, and magnesium, with attention to both their prophylactic and therapeutic roles. Consideration is also given to the safety and efficacy of amiodarone in the postoperative thoracic population. This case report underscores the complexity of managing POAF after anatomic lung resection and highlights the need for greater development, recognition, and implementation of evidence-based guidelines driven by data in pulmonary as opposed to solely cardiac populations. While pharmacologic interventions such as amiodarone have demonstrated effectiveness based on the literature review and were well-tolerated in this case, individualized patient risk assessment remains essential.

## Introduction

Postoperative atrial fibrillation (POAF) is a common complication following noncardiac thoracic surgery, particularly in patients after a pulmonary lobectomy. POAF occurs within seven days of surgery or before hospital discharge and is most commonly seen within the first two to five days [1,2]. The incidence of POAF differs based on the extent of lung resection, with lower rates observed in wedge resections (2%), moderate rates in lobectomies (10%), and the highest rates in pneumonectomies (>20%) [3]. Several prior studies have discussed POAF as not an independent pathology but rather a transient event triggered by surgical and physiologic stressors, though the precise mechanism is often unclear [4-6]. As a result, determining whether it should be managed similarly to spontaneous atrial fibrillation (AF) or require a distinct approach has not been well defined in the literature [1,7].

Effective management of POAF is critical as its occurrence is associated with increased morbidity and mortality, prolonged hospital stays, and higher healthcare costs [8-13]. While guidelines exist for the treatment of POAF, the evidence quality and lack of consistency in the treatment approach highlight a gap in the standard of care consensus [1,13,14]. Therefore, there is a clear need for further development and validation of protocols specific to thoracic surgery to optimize the treatment of POAF in patients who undergo pulmonary lobectomy.

The pathophysiology of POAF following pulmonary lobectomy involves a combination of predisposing patient characteristics, intraoperative stressors, and postoperative inflammatory responses [3]. Key risk factors include advanced age, prior history of AF, elevated CHA2DS2-VASc scores, and extent of lung resection [9-12,15]. This interplays with surgical factors, including intrathoracic manipulation and autonomic nervous system imbalances [12].

Current pharmacologic management of POAF includes rate control strategies such as beta-blockers, calcium channel blockers, and digoxin, as well as rhythm control agents like amiodarone, alongside electrolyte replacement. Non-pharmacologic interventions include electrical cardioversion, optimized fluid balance, and enhanced recovery protocols [10,13,16]. The role of anticoagulation in this population is complex, as postoperative bleeding risk often outweighs the risks of potential thromboembolic complications, such as strokes [3,11,17].

This descriptive case report and literature review will explore POAF with a focus on pharmacologic management strategies in the unique group of pulmonary lobectomy patients. While the case report highlights a segmentectomy patient, it conveys the clinical relevance of this condition in patients who have undergone thoracic surgery procedures.

## Case presentation

A 77-year-old man with a history of malignant melanoma in remission, hypertension, hyperlipidemia, and GERD was evaluated for a right lower lobe (RLL) indeterminate pulmonary nodule. He was a former smoker with prior surgeries including melanoma excision, hiatal hernia repair, and inguinal hernia repair. He had no history of bleeding or cardiac disease and was not on anticoagulation. There had been no prior assessment of cardiac structure, and preoperative cardiac workup was limited to an unremarkable EKG per surgical risk stratification guidelines.

**Figure 1:**
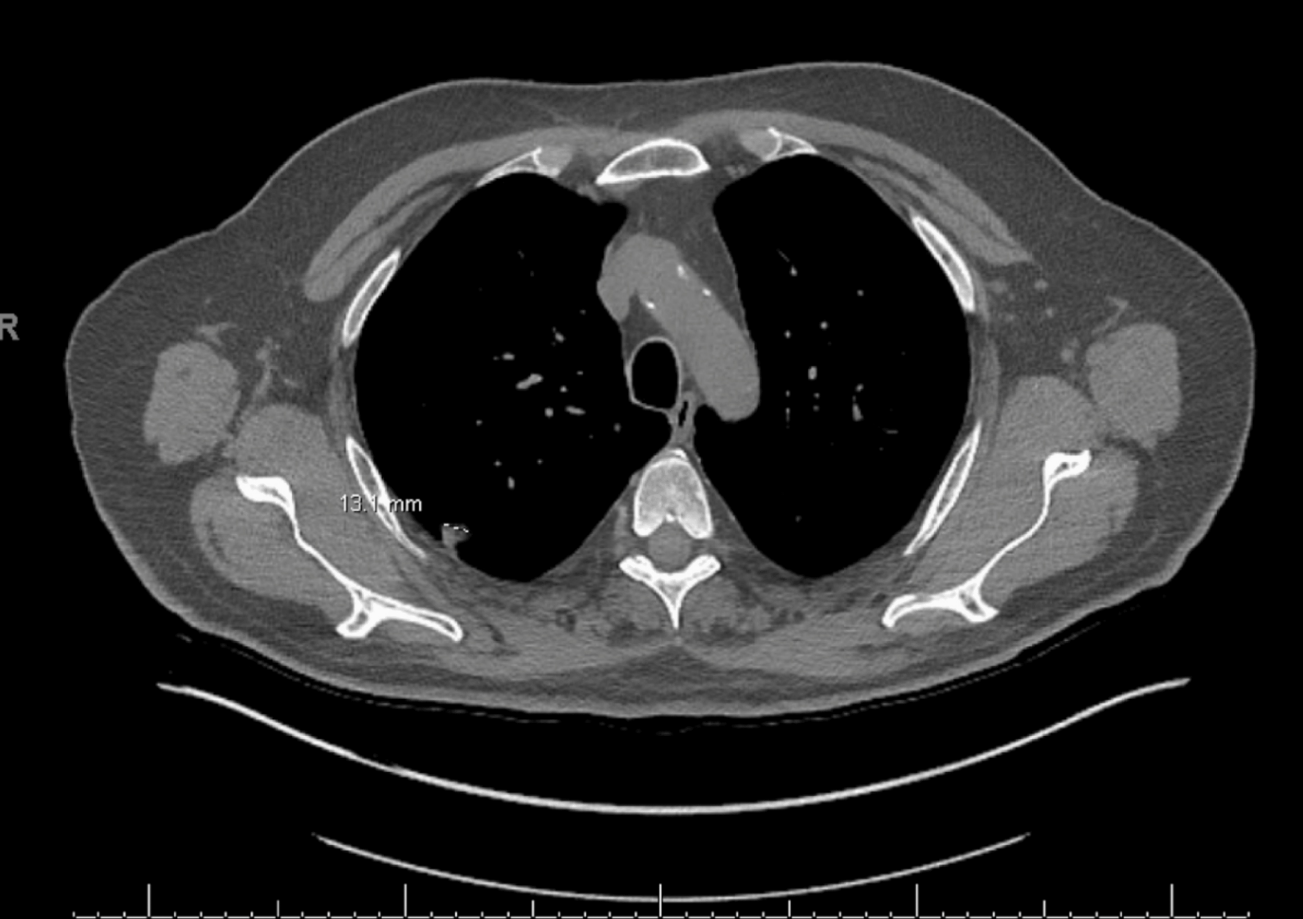
Preoperative chest CT demonstrated a 1.3 cm pulmonary nodule in the RLL, without mediastinal adenopathy or pleural effusion. CT, computed tomography; RLL, right lower lobe

**Figure 2:**
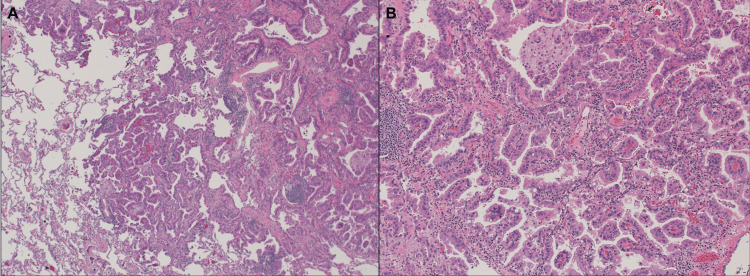
Lung adenocarcinoma. A. Low-power view (H&E, 10×) shows adenocarcinoma involving benign lung parenchyma. B. High-power view (H&E, 40×) shows adenocarcinoma with papillary features and intracellular mucin. The tumor is classified as NSCLC, consistent with moderately differentiated acinar-pattern adenocarcinoma. Immunohistochemistry revealed the absence of PD-L1 expression in tumor cells. NSCLC, non-small cell lung carcinoma

The pulmonary nodule had increased in size over a 12-month period, as shown in the preoperative CT scan (Figure 1). Biopsy results demonstrated non-small cell lung cancer (NSCLC), specifically a moderately differentiated adenocarcinoma with acinar features (Figure 2). The tumor was negative for PD-L1 expression.

The patient underwent uniportal video-assisted thoracoscopic surgery (VATS) basilar segmentectomy and mediastinal lymphadenectomy. Intraoperatively, pneumopexy was performed at the superior segment (S6) of the RLL, along with mediastinal and hilar lymphadenectomy. A right-sided chest tube was placed intraoperatively. Pathological examination revealed invasive adenocarcinoma with negative mediastinal and hilar lymph nodes. All margins were negative for tumor, indicating an R0 resection. 

The patient was asymptomatic in the immediate postoperative period and did not require supplemental oxygen. A chest x-ray obtained in the post-anesthesia care unit (PACU) demonstrated a small right apical pneumothorax, so the chest tube placed intra-operatively was placed on suction with an air leak on postoperative day (POD) 0. The small pneumothorax persisted but remained unchanged throughout the patient’s hospitalization. On POD 1, the patient was started on deep vein thrombosis (DVT) prophylaxis with enoxaparin. A chest x-ray obtained the same day revealed new right chest wall subcutaneous emphysema, though the patient remained asymptomatic. 

**Figure 3:**
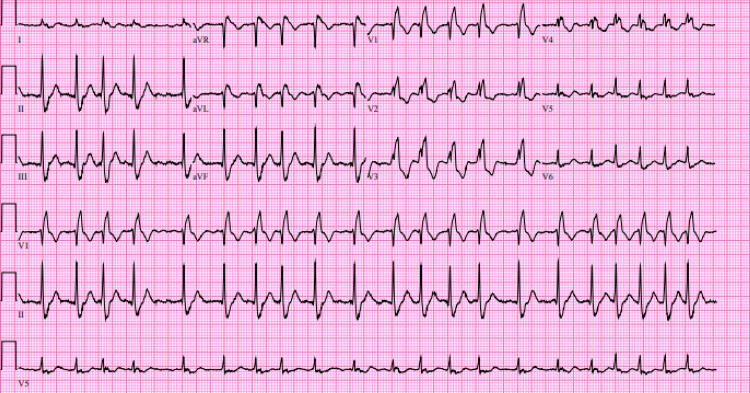
Postoperative EKG demonstrating AF with RVR. 12-lead EKG showing an irregularly irregular rhythm with no identifiable P-waves and a ventricular rate of approximately 130 beats per minute. EKG, electrocardiogram; RVR, rapid ventricular response; AF, atrial fibrillation

On POD 2, the patient developed new-onset AF with rapid ventricular response (RVR), with heart rates up to 150 beats per minute (Figure 3). Metoprolol was considered for rate control but ultimately deferred due to systolic blood pressures in the 90s. Instead, the patient was treated with 2 g of intravenous (IV) magnesium sulfate and a 150 mg bolus of IV amiodarone followed by a 24-hour amiodarone infusion 1.8 mg/mL at 1 mg/min for 6 hours and then 0.5 mg/min for 18 hours, with return to normal sinus rhythm (NSR). Throughout the hospital course, the patient remained asymptomatic during episodes of AF.

**Figure 4:**
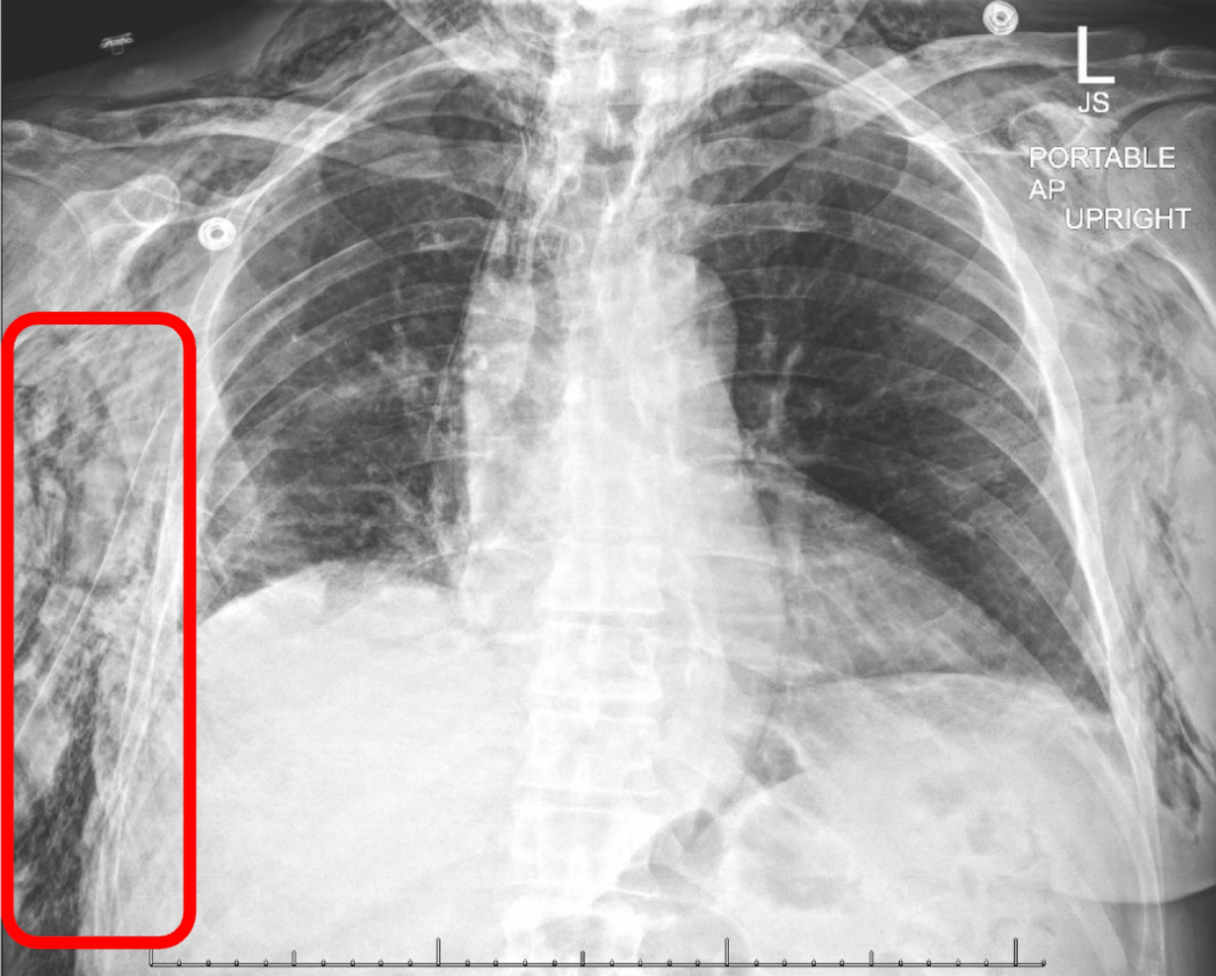
Chest x-ray obtained on POD 5. Demonstrates diffuse subcutaneous emphysema, as indicated by the red box. A small right apical pneumothorax is present and has remained unchanged throughout hospitalization. A right lateral approach chest tube is in place. POD, postoperative day

Daily chest x-rays were obtained throughout the patient’s postoperative course, demonstrating increasing subcutaneous emphysema (Figure 4). The patient remained asymptomatic from the subcutaneous air until POD 5 when he developed facial swelling and a supplemental oxygen requirement. On POD 6, he underwent a bedside cutaneous blowhole procedure at the base of the right neck, and his symptoms progressively resolved throughout the rest of his hospitalization.

The patient continued to have multiple recurrences of AF with RVR throughout the next several days, which were treated with the same regimen as his initial episode. However, following the completion of the amiodarone infusion on POD 7, the patient was transitioned to oral amiodarone 400 mg twice daily. This oral amiodarone, however, was discontinued the following day due to the concern of pulmonary toxicity in a recent pulmonary resection patient. The patient continued to have paroxysms of AF until POD 9, which all resolved without further intervention. There was no air leak on POD 9, and the blowhole VAC dressing was removed. Further, a bedside transthoracic echocardiogram (TTE) was performed on POD 9, which showed grossly normal cardiac function. 

Cardiology was consulted during this patient’s postoperative course, and the management described above was based on their recommendations. Other recommendations by their team included consideration of beta-blockers or calcium-channel blockers for rate control if blood pressures permitted, but the patient’s blood pressures remained too labile for these medications to be used. Administration of Class I anti-arrhythmic medications was not recommended. Long-term anticoagulation was recommended based on a calculated CHADS-VASc score of three, and outpatient cardiology follow-up was scheduled. 

The patient was discharged on POD 11 in NSR. Upon discharge, he was provided with a ZioPatch monitor (iRhythm Technologies, Cypress, CA) for 14 days of monitoring and started on rivaroxaban 20 mg daily. As the etiology of the AF was likely due to surgical stressors and anticipated to be transient, he was not discharged on rhythm or rate control medications, given his return to NSR following surgical recovery.

The patient returned one week after discharge in AF with RVR. He was treated with a one-time IV diltiazem 25 mg bolus, resulting in adequate rate control. Outpatient oral diltiazem 60 mg twice daily was initiated, and the patient’s previous rivaroxaban 20 mg daily was continued. 

Upon outpatient follow-up six weeks after discharge, analysis of the ZioPatch data demonstrated a predominant underlying NSR, with four episodes of AF over 14 days, the longest episode lasting 33 minutes. The patient’s overall AF burden was less than 1%. Given this low burden of AF, the oral diltiazem was discontinued. The patient remains on rivaroxaban 20 mg daily due to a CHADS-VASc score of three. A TTE performed in the outpatient setting revealed a left ventricular ejection fraction of 55-60%, mild aortic root dilatation measuring 4.4 cm, and no other significant abnormalities.

## Discussion

Methods

We performed a retrospective chart review to produce a case report. The patient provided written consent. SCARE (Surgical CAse REport) guidelines were reviewed and followed. Our institution does not require IRB approval for an isolated case report. 

Our literature review was conducted to identify studies evaluating pharmacologic treatment strategies for POAF following pulmonary lobectomy. The search was performed in Scopus and PubMed using search criteria and terms involved permutations of “atrial fibrillation,” “cardiac arrhythmia,” “postoperative,” “pulmonary resection,” and “lobectomy” with exclusion parameters for non-human studies. The search was performed on 03/25/2025 and included articles from the past 30 years. This was conducted with the assistance of a reference librarian. 

To ensure thorough coverage of the literature, reference lists of selected articles were reviewed for additional sources. The resulting collection of articles was screened by two independent reviewers. 

Studies were included if they involved adult patients who developed new-onset POAF following pulmonary lobectomy, encompassing both open and minimally invasive surgical approaches. Eligible studies specifically evaluated pharmacologic treatment strategies for POAF and included randomized controlled trials (RCTs), cohort studies, case-control studies, systematic reviews, and meta-analyses. Case series with at least 10 patients were also included, as data on pharmacologic management of POAF following lobectomy remain limited; smaller series can provide valuable insight into treatment patterns and clinical decision-making in this specific surgical population, especially when larger studies are lacking. 

Studies were excluded if they focused on non-pulmonary lobectomy surgeries without separately reported data for lobectomy patients, examined pre-existing AF rather than new-onset POAF, or did not evaluate any treatment strategies for POAF. Additional exclusions included case reports with fewer than 10 patients, narrative reviews, editorials, expert opinions, and other non-peer-reviewed literature. Studies solely discussing postoperative atrial flutter, those involving non-human subjects, or those not published in English were also excluded. Any disagreements during the screening process were resolved through discussion between the two reviewers until a consensus was reached.

Diagnostic Criteria 

The diagnostic criteria for POAF following pulmonary lobectomy rely on both electrophysiologic and clinical definitions. According to the American Association for Thoracic Surgery (AATS), POAF is identified by electrocardiographic (ECG) evidence of AF, marked by the absence of discrete P waves, irregularly irregular RR intervals, and fibrillatory waves, lasting at least 30 seconds or for the duration of the ECG recording if shorter [14]. 

Clinically significant POAF is defined by the AATS as AF occurring intraoperatively or postoperatively that requires rate or rhythm control, anticoagulation, and/or results in prolonged hospitalization. Symptoms may include hypotension, dizziness, reduced urine output, or fatigue; however, asymptomatic episodes also meet the diagnostic threshold if confirmed by ECG and at or above duration criteria [14]. 

In clinical studies involving lung resection, including lobectomy, POAF is typically defined as any ECG-confirmed episode of AF, symptomatic or asymptomatic, occurring within seven days post-surgery or before hospital discharge [2]. Both electrophysiologic and clinical criteria should be documented in the medical record and reported in research, as recommended by the AATS [14].

Postoperative prophylaxis

Amiodarone

Evidence suggests that amiodarone used as primary prevention reduces the incidence of POAF. Riber et al. conducted an RCT involving 254 patients, which demonstrated that while amiodarone significantly decreased the incidence of POAF in lung cancer patients undergoing thoracic surgery, it did not impact hospital length of stay or cost [18]. Phillips et al. reported a statistically significant reduction in POAF using a prophylactic regimen of a 300 mg IV bolus followed by 400 mg oral amiodarone three times daily for three days; however, this study was based on a historical comparison rather than a prospective control group, limiting the strength of its conclusions [19]. In contrast, Khalil et al. performed a prospective clinical trial that showed a significant reduction in POAF among patients receiving prophylactic amiodarone compared to controls [20]. Supporting these findings, the 2023 AATS guidelines classify prophylactic amiodarone as a Class IIa recommendation in intermediate- and high-risk patients undergoing lung resection [14]. Taken together, these studies highlight amiodarone as a potentially effective yet underutilized strategy for reducing POAF in this population.

Beta-Blockers

There is limited evidence supporting the efficacy of beta-blockers specifically for the prophylaxis of POAF in thoracic surgery patients. However, there is more robust evidence regarding the risks associated with beta-blocker withdrawal. Studies have shown that patients who discontinue beta-blockers may experience higher levels of circulating catecholamines compared to those not on beta-blockers [21]. Reflecting this, the AATS guidelines emphasize the increased risk of POAF following beta-blocker withdrawal and recommend that patients who are chronically on beta-blockers continue them in the postoperative period [14]. This distinction highlights the need for further research into beta-blockers as primary prophylaxis, while reinforcing the importance of avoiding abrupt discontinuation in at-risk patients.

Calcium Channel Blockers

A large-scale RCT demonstrated that diltiazem decreases the incidence of POAF in patients undergoing major thoracic surgeries [22]. However, specific data demonstrating the effectiveness of diltiazem to prevent POAF following lung resection are limited. 2014 AATS guidelines, however, state that it is reasonable to administer diltiazem to those patients with preserved cardiac function who are not taking beta-blockers preoperatively in order to prevent POAF [14].

Magnesium

Preoperative administration of magnesium sulfate has been associated with a decreased incidence of POAF in non-cardiac thoracic surgery [23-25]. However, high-quality, procedure-specific data, particularly in patients undergoing lung resection, remain limited. Most available studies do not stratify outcomes by type of thoracic surgery, making it difficult to draw definitive conclusions for the lung resection population specifically. Further research is needed to determine optimal dosing, timing, and patient selection in this subgroup.

Postoperative abortive therapy

Amiodarone

While amiodarone is widely used for rhythm control in POAF, its association with pulmonary toxicity warrants particular attention in patients undergoing lung resection who have existing lung pathology or limited pulmonary reserve [26-28].

Berry et al. 2014 performed a retrospective analysis of 1,412 patients undergoing major lung resection (lobectomy, bilobectomy, or pneumonectomy) and investigated the relationship between amiodarone and the incidence of respiratory complications [29]. Within the cohort, POAF developed in 232 (16%) patients. The study excluded patients who developed AF after a respiratory complication to isolate the impact of amiodarone on pulmonary outcomes. Among patients with POAF, 50% received amiodarone. Importantly, its use was evenly distributed across groups with similar baseline pulmonary function and surgical extent. The incidence of respiratory complications did not significantly differ between treatment arms, suggesting that the administration of amiodarone in this context does not confer a significantly increased risk of pulmonary complications. This emphasizes its utility as an antiarrhythmic agent in lung resection patients without preexisting pulmonary compromise. However, the retrospective nature of this study introduces the potential for selection bias and unmeasured confounding. 

Two prospective studies further support the short-term safety and efficacy of amiodarone in the postoperative setting. In the first of 160 patients undergoing various lung resections for NSCLC, 13% developed POAF. All were treated with IV amiodarone at a loading dose of 5 mg/kg (equivalent to 350 mg for a 70 kg adult) over 30 minutes, followed by a maintenance dose of 15 mg/kg (equivalent to 1,050 mg for a 70 kg adult) over 24 hours. Sinus rhythm was successfully restored in 91% (20 of 22) of treated patients, with no reported adverse effects [30]. A second prospective study of 250 pulmonary resection patients reported a 22% incidence of postoperative supraventricular arrhythmias, predominantly AF (88%). Using the same dosing protocol, amiodarone restored sinus rhythm in 86% of cases. While six patients were hemodynamically unstable and required electrical cardioversion, pharmacologic treatment alone was effective in most. Bradycardia occurred in 14% of patients, but no cases of heart failure or amiodarone-related mortality were reported [31]. 

Together, these findings support the efficacy and tolerability of amiodarone for short-term postoperative use, which similarly has a low risk of pulmonary complications. In contrast, long-term amiodarone therapy, over months to years, is associated with a significantly higher risk of pulmonary fibrosis and interstitial lung disease [27,32-34]. However, the long-term risks of pulmonary complications, including delayed pulmonary toxicity, following short-term amiodarone treatment are not well studied. Therefore, there is a necessary role for monitoring strategies after discharge, particularly in patients with reduced pulmonary reserve, to detect delayed pulmonary toxicity. 

In summary, short-term amiodarone use appears to be a safe and effective option for rhythm control of POAF in patients undergoing lung resection. Its high conversion rate to sinus rhythm, manageable side effect profile, and low short-term pulmonary risk make it especially useful in elderly patients and those undergoing major resections such as lobectomy or pneumonectomy. Nonetheless, the current body of evidence largely stems from small or non-randomized studies. While the available data are encouraging, larger RCTs are needed to confirm these findings and guide long-term management.

Beta-Blockers

Beta-blockers are also first-line agents to achieve rate control in patients who develop AF after cardiac surgery unless contraindicated or ineffective [14,35,36]. In such cases, nondihydropyridine calcium channel blockers are considered as alternatives. The optimal heart rate target depends on the clinical setting, symptom burden, presence of heart failure, and whether rate control is used alone or alongside rhythm control. In hemodynamically stable patients, lenient rate control, defined as a resting heart rate <110 beats per minute, has been shown to be non-inferior to stricter control in terms of clinical outcomes, based on the RACE II trial and post-hoc analyses from the AFFIRM and RACE studies. Therefore, lenient rate control is a reasonable initial strategy, with stricter targets reserved for patients with persistent symptoms or suspected tachycardia-induced cardiomyopathy [35,37-39]. 

Evidence guiding the use of beta blockers for managing POAF specifically in lobectomy patients is limited, and most recommendations are extrapolated from studies involving broader thoracic surgery populations. While similar, it is important to recognize the differences in the physiological and surgical interplay that exist in cardiac surgery data when compared to pulmonary procedures. This somewhat limits the direct applicability of cardiac surgery data to the management of AF after lobectomy. 

Metoprolol and esmolol are among the most commonly used beta-blockers in the acute management of POAF, largely in part due to their wide availability, intravenous formulations, and cardioselective beta-1 receptor activity [35,40]. Metoprolol is more frequently prescribed because of its longer duration of action and widespread clinical familiarity. In contrast, esmolol has an ultrashort half-life, making it especially useful in hemodynamically unstable or rapidly evolving postoperative settings where close titration is needed. Both agents are included as Class I recommendations for rate control in the AATS guidelines [14].

Landiolol is a short-acting, ultra-selective β₁-adrenergic blocker that has been shown to be approximately eight times more β₁-selective than esmolol, with a half-life of 4 minutes [40]. Administered at low doses via continuous infusion, landiolol has been found to effectively reduce heart rate and facilitate the restoration of sinus rhythm in patients who develop POAF following lung resection procedures. In a prospective study by Nojiri et al., landiolol demonstrated both efficacy and safety in controlling postoperative arrhythmias without causing significant hypotension or bradycardia [41]. Similarly, Nakano et al. reported favorable outcomes with landiolol infusion, highlighting its rapid onset, short half-life, and minimal negative inotropic effects, which make it particularly suitable for the immediate postoperative setting where hemodynamic stability is critical [42]. Access to landiolol remains limited in the United States, as it was only recently approved by the FDA and is not yet widely available, despite evidence supporting its cost-effectiveness in European settings [43]. Even in institutions where it is available, its use may be restricted by local protocols or a lack of clinical familiarity.

Calcium Channel Blockers

Diltiazem, a nondihydropyridine calcium channel blocker, is often employed as a third-line agent for rate control in hemodynamically stable patients with POAF who are not candidates for or do not respond to beta-blockers. Its use in the setting of thoracic surgery, particularly lung resection, has been studied less extensively than amiodarone or beta blockers, but it remains a relevant therapeutic option due to its ability to reduce atrioventricular (AV) nodal conduction and control ventricular rate. 

In a prospective randomized trial by Bobbio et al., patients who developed POAF following pulmonary resection, including pneumonectomy, bilobectomy, lobectomy, and segmentectomy, were randomized to receive either amiodarone or diltiazem. The study found no significant difference between the two groups in terms of time to conversion to sinus rhythm, length of hospital stay, or complication rates, suggesting that diltiazem may be as effective as amiodarone in select postoperative patients. However, the small sample size of 30 patients and the limited scope of the study restrict the generalizability of its findings [16]. 

While diltiazem has demonstrated some utility in POAF management, its role remains adjunctive and is largely used in patients who are intolerant to beta-blockers, nonresponsive to beta-blockers, or have a history of severe reactive airway disease or asthma. Larger studies comparing calcium channel blockers to other agents, particularly in the context of lung resection and varying degrees of pulmonary reserve, are needed to better define their safety and efficacy in this population.

Class I Antiarrhythmics

Class 1 antiarrhythmic drugs, specifically Class IC agents such as flecainide and propafenone, may be considered for pharmacologic cardioversion and maintenance of sinus rhythm in carefully selected patients with POAF after lobectomy. Their use is limited to patients without a history of myocardial infarction, coronary artery disease, significant left ventricular hypertrophy, impaired left ventricular function, or moderate or greater valvular heart disease. According to AATS guidelines, these agents should be co-administered with an AV nodal blocking agent to mitigate the risk of 1:1 atrial flutter and rapid ventricular conduction. Due to their proarrhythmic potential, Class IC agents are contraindicated in patients with structural heart disease [14].

The Society of Thoracic Surgeons recommends flecainide as a preferred agent for chemical cardioversion in patients with POAF following pulmonary resection, particularly in those with significant pulmonary dysfunction or after pneumonectomy, due to the potential for amiodarone-induced pulmonary toxicity in this population [44]. However, amiodarone remains a commonly used agent for POAF in patients without significant pulmonary risk, as the incidence of amiodarone-induced acute respiratory distress syndrome (ARDS) appears to be higher after pneumonectomy than lobectomy [14]. 

While Class I antiarrhythmic drugs are recommended by current guidelines for rhythm control in POAF, their use in the immediate postoperative period after lobectomy is often limited by concern for proarrhythmia and hemodynamic instability. Given that POAF is frequently transient and related to surgical stressors, initial treatment typically focuses on rate control and management of reversible causes. Rhythm control with Class I agents is reserved for selected stable patients without structural heart disease, balancing efficacy with safety. However, in many institutions, these medications are not routinely initiated by surgeons due to limited familiarity and comfort with their use, and prescription may be limited to cardiology or electrophysiology services.

Other Antiarrhythmics

The 2014 AAST guidelines recommend IV digoxin for rate control in POAF patients with heart failure, left ventricular dysfunction, or hypotension [14]. However, its use in lobectomy patients lacks direct evidence and is largely extrapolated from cardiac surgery populations. A key limitation of digoxin in the acute postoperative setting is its delayed onset of action, particularly in the context of elevated sympathetic tone. This concern regarding a slower onset of action in states of elevated catecholamines is well supported by pharmacological principles and clinical guidelines. The American Heart Association, American College of Cardiology, and Heart Rhythm Society note that IV digoxin has an onset of action exceeding one hour, with a peak effect at approximately six hours. This makes it suboptimal for rapid rate control in acute settings, especially when sympathetic tone is high [45]. This slower onset is attributed to digoxin’s reliance on increased vagal tone to exert its AV nodal effects, which is often counteracted by high circulating catecholamines that enhance sympathetic activity and diminish vagal responsiveness [46]. To date, no trials have specifically evaluated digoxin use in POAF following lobectomy, and current recommendations are derived primarily from studies of POAF after coronary artery bypass graft surgery [47].

Comparative Effectiveness of Rate and Rhythm Control

There is a notable lack of high-quality evidence guiding the management of POAF in non-cardiac thoracic surgery patients, such as those undergoing lobectomy. Most available data is derived from cardiac surgery cohorts, which differ significantly from lobectomy populations in terms of comorbidities, physiologic stress, and perioperative risk. This limits the generalizability of findings. Methodological heterogeneity and inconsistent outcome definitions further complicate the extrapolation of results [48,49]. Importantly, no clear advantage has been demonstrated between rate and rhythm control strategies for POAF after thoracic surgery, and robust comparative data such as attributable risk reduction (ARR) and number needed to treat (NNT) are lacking. The AATS highlights this as a key evidence gap, noting the absence of randomized trials specific to thoracic surgery patients. Existing studies are underpowered and primarily limited to cardiac cohorts, leaving lobectomy-specific data largely unexplored [14,48,50]. 

The largest randomized trial in cardiac surgery patients (n=523) found no significant difference between rate and rhythm control in terms of the primary outcome, hospital days within 60 days (median 5.1 vs. 5.0 days; P=0.76), nor in mortality or persistent AF at 60 days (84.2% vs. 86.9% free from AF; P=0.41) [48]. Similarly, a recent meta-analysis of randomized trials and observational studies found no significant difference between strategies in AF recurrence, mortality, or length of stay (e.g., AF recurrence within one week: risk ratio 1.1, 95% CI 0.6-1.9) [49].

Guidelines and protocols

The most recent AATS and Enhanced Recovery After Surgery (ERAS) guidelines recommend a risk-stratified approach for the prevention and management of POAF in thoracic surgery, including pulmonary lobectomies. This section provides a concise summary of these recommendations.

To contextualize the recommendations presented in this case report and narrative review on POAF following lobectomy, it is important to outline the classification system commonly employed by the AATS. A Class I recommendation indicates strong evidence or general consensus that an intervention is beneficial, useful, and effective, and therefore, should be performed. A Class IIa recommendation suggests that the intervention is reasonable to perform, with moderate supporting evidence. A Class IIb designation reflects weaker evidence or agreement, indicating that the intervention may be considered. In contrast, a Class III recommendation signifies that the intervention is not beneficial and may potentially be harmful. These classes are accompanied by levels of evidence, ranging from Level A (derived from multiple randomized clinical trials or meta-analyses) to Level C (based on expert opinion, case studies, or standard clinical practice). This structured approach supports the interpretation and application of clinical recommendations related to the management of POAF [14,51].

Prevention strategies include a Class I recommendation to continue beta-blockers perioperatively to prevent withdrawal and a Class IIb recommendation for IV magnesium administration when deficiency is suspected. For patients deemed intermediate- to high-risk of developing POAF, IV amiodarone and diltiazem are both Class IIa options. Atorvastatin (Class IIb) may also be considered in statin-naïve patients. Though not traditionally used for rate or rhythm control, atorvastatin may play a preventative role by attenuating systemic inflammation, as evidenced by its ability to lower C-reactive protein (CRP) levels, an inflammatory marker associated with increased risk of AF [52].

In high-risk cardiac surgery patients with a history of AF who are not candidates for long-term anticoagulation, intraoperative left atrial appendage (LAA) excision is a Class IIb recommendation for stroke risk reduction. The Society of Thoracic Surgeons guidelines support LAA management (excision, occlusion, or exclusion) in such settings due to its association with fewer thromboembolic events [53]. However, routine prophylactic LAA excision is not recommended during non-cardiac thoracic surgeries such as lung resections. It is generally reserved for select cases where the LAA is directly involved by tumor or when pre-existing AF is present with contraindications to anticoagulation. In rare cases of locally advanced lung cancer, partial LAA resection may be performed as part of an extended resection to achieve oncologic control, but such procedures carry increased risk [54].

For hemodynamically unstable POAF, urgent synchronized cardioversion is recommended. If POAF persists beyond 48 hours, anticoagulation is generally recommended; however, this threshold should not be viewed as absolute. The decision to initiate anticoagulation should be individualized based on stroke risk, bleeding risk, and shared decision-making. For stable patients, a rate control strategy is generally preferred, though rhythm control may be appropriate for refractory or symptomatic cases. Patients with RVR should receive IV beta-blockers (e.g., esmolol and metoprolol) or calcium channel blockers (e.g., diltiazem and verapamil), with caution in those with hypotension or heart failure. Combination therapy or IV amiodarone may be considered if monotherapy is inadequate. Combination therapy is a Class IIa recommendation and is the concurrent use of multiple AV nodal blocking agents, including beta-blockers, non-dihydropyridine calcium channel blockers, or digoxin. This approach may help achieve adequate rate control when a single agent is insufficient. The choice of agents and dosing should be individualized, with close monitoring to avoid bradycardia [14]. 

Pharmacologic cardioversion is a reasonable option for symptomatic, hemodynamically stable POAF, with IV amiodarone commonly used. Maintenance of sinus rhythm can be achieved with antiarrhythmic agents such as amiodarone, sotalol, flecainide, propafenone, and dofetilide, depending on patient comorbidities. Flecainide and propafenone (Class I agents) are recommended for rhythm control in selected patients without structural heart disease, but their use in the immediate postoperative period is often limited due to concerns about proarrhythmia and hemodynamic instability. Sotalol carries a proarrhythmic risk and requires careful initiation with cardiac monitoring, while dofetilide use is restricted due to its requirement for inpatient initiation and close renal function monitoring because of its renal clearance. Additionally, dronedarone is contraindicated in heart failure [14,51]. Importantly, there is a lack of dedicated evidence guiding antiarrhythmic therapy specifically in lobectomy patients, highlighting the need for focused clinical studies and guideline development in this population. 

In April 2024, the ERAS Cardiac Society introduced a standardized POAF prevention and management order set for cardiac surgery [51]. While these recommendations emphasize beta-blockers, antiarrhythmic agents, and electrolyte optimization, their application to thoracic surgery remains unclear. Differences in comorbidities, surgical approach, postoperative hemodynamics, and arrhythmia profiles limit the generalizability of these protocols to patients undergoing pulmonary resections. Notably, lobectomy patients, who represent a distinct surgical cohort with unique perioperative considerations, remain underrepresented in POAF research. This underscores a critical gap in the evidence base and highlights the need for thoracic-specific POAF guidelines tailored to this population.

## Conclusions

This case highlights the complex management of POAF in thoracic surgery patients, particularly following pulmonary resection. Hemodynamic instability limited the use of first-line rate-controlling agents such as beta-blockers and calcium channel blockers. As a result, amiodarone was initiated despite concerns for pulmonary toxicity, an especially relevant risk after lung resection. Though amiodarone is supported by literature as both a prophylactic and therapeutic agent, its role varies by timing and clinical context, and its use remains limited in practice due to safety concerns and institutional variability. Furthermore, many of the studies in the literature are limited by size and retrospective design. Class I antiarrhythmics were not considered due to their proarrhythmic profile and general unfamiliarity among surgical teams. Ultimately, the therapeutic approach in this case was necessarily reactive, shaped by evolving hemodynamics and constrained pharmacologic options. When the patient re-presented in AF with RVR and blood pressure had stabilized, diltiazem was initiated for rate control. This stepwise escalation illustrates the need for dynamic, individualized care pathways for thoracic surgery patients in the immediate postoperative setting. Importantly, this case underscores the limitations of existing POAF guidelines, particularly when applied to thoracic patients with unique cardiopulmonary vulnerabilities. Risk stratification tools such as CHA₂DS₂-VASc were not designed for transient, surgery-related AF and may fail to accurately assess thromboembolic risk in this context. Multidisciplinary input is essential, especially as patients are often discharged on anticoagulation without a clear plan for rhythm monitoring or long-term antiarrhythmic therapy. Although current guidelines recommend anticoagulation for POAF, the optimal duration and criteria for initiation remain controversial. Future thoracic-specific guidelines should incorporate variables such as symptom burden, AF recurrence risk, and degree of postoperative cardiopulmonary compromise to guide management. Ultimately, this case contributes to the growing call for targeted research and dedicated POAF protocols in thoracic surgery to support proactive, evidence-based decision-making in this high-risk population.
